# Retinal vein occlusion and the use of a dexamethasone intravitreal implant (Ozurdex®) in its treatment

**DOI:** 10.1007/s00417-016-3350-x

**Published:** 2016-05-13

**Authors:** Justus G. Garweg, Souska Zandi

**Affiliations:** Berner Augenklinik am Lindenhofspital, University of Bern, Swiss Eye Institute, Luzerner Strasse 1, CH-6343 Rotkreuz, Bern Switzerland

**Keywords:** Retinal vein occlusion, Central retinal vein occlusion, Branch retinal vein occlusion, Pathophysiology, Dexamethasone, Intravitreal implant, Mead, Copernicus, Galileo, Geneva, Score, Shasta, Review of literature

## Abstract

**Purpose:**

To review published data pertaining to the clinical experience with a dexamethasone intravitreal implant (Ozurdex®) with a view to establishing a clinically based therapeutic regime.

**Methods:**

A PubMed search using the MeSH terms “retinal vein occlusion” and either “pathophysiology” or “dexamethasone intravitreal implant” was undertaken for manuscripts published until August 2015. The analysis included studies involving minimally 15 patients under a prospective design or 30 under a retrospective design, a minimal follow up of 6 months, and at least 2 intravitreal Ozurdex® injections per eye.

**Results:**

In the vast majority of eyes, satisfactory outcomes were achieved with retreatment intervals of between 3 and 5 months. Initial evidence indicates a similar efficacy compared to anti-VEGF therapies as a first-line treatment. Safety concerns associated with the long-term and repeated use of Ozurdex® are not borne out by clinical findings: its implantation is not associated with a sustained increase in intraocular pressure (IOP) over time or with the number of applications.

**Conclusion:**

Compared with anti-VEGF therapies, the burden of retreatment is reduced. In patients with chronic macular edema not responsive to repetitive anti-VEGF therapies, the outcome after dexamethasone implant treatment is encouraging. However, these results are achieved at the expense of side effects typically associated with steroids: in up to 20 % of the Ozurdex®-treated patients, an elevation in IOP, which could be medically controlled in the majority of cases, and cataract formation or progression was observed.

## Introduction

Within the last 10 years, intravitreal pharmacotherapy has revolutionized the therapeutic options for macular edema (ME)-associated retinal vascular diseases, and particularly for retinal vein occlusion (RVO). Substantial improvements, both visual and morphological, have been achieved under treatment with anti-VEGF agents and corticosteroids. Nevertheless, the efficacy of these intravitreal agents and their long-term outcome is still evolving. Moreover, although anti-VEGF agents can elicit a rapid regression of neovascularization, photocoagulation with a scatter laser still represents the standard of care in the prevention of extrafoveal neovascular complications whereas it remains controversial for prophylactic treatment [1]. Data that have been gleaned from randomized clinical trials regarding intravitreal therapy with ranibizumab and dexamethasone reveal significant functional improvements as compared to those that are achieved solely using laser therapy in the treatment of branch retinal vein occlusion (BRVO) and central RVO (CRVO). For the treatment of ME following RVO, intravitreal aflibercept has also been approved. However, more accurate head-to-head studies are needed to assess the relative efficacies of licensed therapies for RVO [2].

## Pathophysiological aspects of the disease

In patients with RVO, ME is the most frequent cause of visual loss, irrespective of whether the macula is or is not perfused. If the visual loss that is associated with neovascularization is left untreated, vitreal haemorrhaging, tractional retinal detachment, or neovascular glaucoma can be expected to ensue in consequence of a breakdown or permanent damage to the blood–retinal barrier resulting in low-grade inflammation and relative ischemia. Therefore, an increase in the production of VEGF-A is provoked, which perpetuates vascular leakage and ME [3]. Not surprisingly, VEGF is an effective target for therapeutic intervention in vascular diseases of the retina, including CRVO [4, 5]. Anti-VEGF therapy has been demonstrated to be effective in the treatment of CRVO in several clinical trials, including CRUISE [4], HORIZON [5], GALILEO [6] and COPERNICUS [7].

The degree of ischemia is correlated with the intravitreal levels of VEGF and with the prognosis [8]. Angiographically, it can be classified according to the area of non-perfusion, nowadays, ideally using ultra-widefield technology [9, 10]. There exists no generally accepted definition for distinguishing between ischemic and non-ischemic RVO. However, from a clinical point of view, RVO may be deemed as non-ischemic (perfused) if fewer than 10 disc areas of non-perfusion are identified angiographically, which represents 75 % to 80 % of all newly diagnosed CRVO cases. The complete loss of retinal capillaries, with late venous staining in fluorescein angiography, is indicative of a significant ischemia-induced up-regulation of VEGF, and is associated with a high risk for secondary complications. If 10 or more disc areas of non-perfusion are implicated, then the risk for neovascularization of the anterior segment is heightened [9, 11]. If in this situation, the vision is good and there is no relevant ME, then anti-VEGF therapy would not be recommended, but the ischemic areas should be subjected to careful and, in the early phase, timely observation and eventually peripheral panretinal laser photocoagulation to avoid the risk of neovascular complications. It is worthy of note that treatment with an anti-VEGF agent can mask the degree of underlying ischemia [1, 2].

Although the mean vitreal levels of VEGF are elevated in both disease states (CRVO and BRVO), in one-third of the eyes, these may fall within the normal range despite the presence of ME [8, 12, 13]. This finding points to the existence of VEGF-independent pathways that can drive ME and could be the reason that a fraction of patients is less responsive to anti-VEGF therapy alone.

RVO is not an isolated retinal vascular disease; choroidal involvement is part of the pathology [14] and pigment epithelial detachment is present in up to one third of the patients.

## The dexamethasone-based intravitreal implant (Ozurdex®)

Ozurdex® is a dexamethasone-bearing vehicle, which has been developed in the form of a biodegradable intravitreal implant and which delivers a 700-μg dose of the drug to the retina and the vitreous. It has been approved for use in the treatment of RVO-associated ME [24] and non-infectious posterior uveitis [16] by the Federal Office of Food and Drug Administration in the USA (FDA), by the Commission for European Medicines Administration (EMA), and by Swissmedic. Ozurdex® has also been used efficaciously in the treatment of other clinical conditions including the postoperative ME that is associated with cataract surgery (Irvine-Gass syndrome) and vitrectomy, diabetic ME, persistent ME, intra-ocular inflammation and adjunctively in cases of age-related macular degeneration [15]. However, randomized clinical trials relating to the use of Ozurdex® in the treatment of these conditions have as yet not all been completed. Safety concerns associated to the use of Ozurdex® include the formation or progression of cataracts and a transient increase in intraocular pressure that peaks after 1 to 2 months, yet is usually amenable to medical management [15, 17, 18].

Recently, it was shown by aqueous laser flare measurements that the impact of Ozurdex® on recovery of the uveovascular barrier in eyes with RVO correlates with its effect on visual acuity and central retinal thickness [19].

## Published data appertaining to the effects of steroids on RVO

In the SCORE study — a multicenter clinical trial — the efficacy and safety of 1-mg and 4-mg doses of preservative-free intravitreal triamcinolone were assessed in patients with ME that had developed secondary to perfused CRVO. This study marked a turning point in the management of RVO, since it was the first of its kind to report on an effective treatment strategy for CRVO-associated ME. For BRVO-associated ME, no differences in visual acuity were observed between the standard care (laser treatment) and the triamcinolone-treated groups. However, the adverse event rates — particularly for rises in intraocular pressure and the formation or progression of cataracts — were higher in the group of BRVO patients that had been treated with 4-mg doses of triamcinolone than in either the BRVO-patient group that had received 1-mg doses of the drug or the standard care one [20, 21].

In the Geneva study — a randomized, controlled, clinical trial — 1267 patients with ME, which had developed secondary to either CRVO (35 %) or BRVO (65 %), were monitored for minimally 12 months after treatment with 0.35- and 0.7-mg doses of dexamethasone [22, 23]. The dexamethasone-treated eyes manifested significant improvements in visual function in contrast to the untreated controls, and they sustained a supportable profile of side effects. A sub-group of 17 patients were monitored for a minimal follow-up period of 50 months. The data that were gleaned from these individuals revealed the visual prognosis to be better in BRVO- than in CRVO-afflicted eyes. In more than 50 % of these patients, a cataract progression was observed. However, a persistent rise in intraocular pressure was reported in only one case; and, likewise, in one case alone, neovascularization and vitreal haemorrhaging were apparent. Hence, treatment with dexamethasone may be deemed safe in the long run [22, 23]. A limitation of the Geneva study was that only two injections of dexamethasone at fixed 6-monthly intervals were administered. During the intervening period, the visual acuity deteriorated and the retinal thickness increased substantially, owing to the subsidence of drug activity. This problem was avoided in eyes that underwent anti-VEGF therapy, since the drug was delivered on a monthly basis in this study [23]. From the body of data that has been collected to date, it is now evident that the effects of intravitreally-administered dexamethasone can be sustained for 4 months (range: 3 to 7 months) irrespective of the patient’s clinical background. A retreatment initiation on an as needed basis (PRN regime) would necessitate reinjection intervals of substantially less than 6 months for the vast majority of eyes [24, 25–27]. Since the recurrence of retinal edema precedes the functional impairment by 2 weeks, the decision for reinjection would ideally be based on OCT criteria [28].

In the Shasta study — a multicenter retrospective clinical trial — 289 patients with CRVO- or BRVO-associated ME were evaluated after treatment with dexamethasone. Two to 9 dexamethasone implants (mean: 3.2) were injected either as the sole therapy (29.1 %) or in conjunction with other treatment strategies. The mean duration of the ME prior to the injection of the first dexamethasone implant was 18.4 months, and the mean re-injection interval was 5 to 6 months. A best-corrected visual acuity increase of +1.0 line was attained 4 weeks after the onset of treatment, and this level was subsequently sustained for 20 weeks after the injection of the final dexamethasone implant. The central retinal thickness decreased significantly compared to the baseline level (*P* ≤ 0.037) and a best-corrected visual acuity increase of ≥ 2 lines was achieved in 66.7 % of the patients with CRVO and in 59.7 % of those with BRVO. In 32.6 % of the CRVO- and BRVO-afflicted eyes, an intraocular pressure increase of ≥ 10 mm Hg was reported. In 29.1 % of the patients, intraocular pressure-lowering medication was administered, and in 1.7 %, incisional glaucoma surgery was called for. In this study, a larger cohort of RVO patients receiving more than two dexamethasone-bearing implants was assessed than heretofore, and no new safety concerns were identified as a consequence of the multiple injections [29, 30]. In an earlier published retrospective assessment of 33 RVO-afflicted eyes, re-treatment with dexamethasone was necessary 4.7 ± 1.1 months after the first injection and 5.1 ± 1.5 months after the second in order to sustain a significant improvement in the best-corrected visual acuity and in the central retinal thickness. No side effects other than those to be expected after the intraocular administration of corticosteroids were revealed [31]. Functional improvements were evident already after the 1st month of treatment with dexamethasone, and were thereafter sustained until the end of the 3rd month; but from the 4th month onward, they were lost. The repeated injection of dexamethasone over a time course exceeding 12 months is deemed to be a safe procedure, even though it is associated with accelerated cataract progression and rises in intraocular pressure which, nevertheless, can usually be brought under control by medical management [26, 32]. In a retrospective study involving a consecutive series of 51 eyes of 49 patients with RVO-associated ME, 70 % of the individuals responded to injections of dexamethasone implants with an improvement in visual acuity and a recession in ME within 3 months of treatment, and in 30 % of the eyes, the gain in visual acuity was ≥15 letters. In 56 % of the patients, a relapse was observed after a median follow-up time of 17 to 18 weeks, and re-injections of dexamethasone were required to restore the gains in visual acuity. However, in some of the eyes, the duration of the positive response to treatment was curtailed to 10 weeks. In 27 % of the eyes, the rises in intraocular pressure needed to be medically controlled, and in 5 %, neovascular complications developed [24]. The findings appertaining to rises in intraocular pressure accord well with the data that are presented in another study, which involved the retrospective analysis of 342 RVO-afflicted eyes, that had been monitored for 8 months after treatment with intravitreal injections of dexamethasone [33]. In 20 % of the eyes, the intraocular pressure increased; in 9 %, it rose by more than 10 mm Hg above the baseline level, but in only 2 % did the rise exceed 35 mm Hg. Eyes in which a pre-existing glaucomatous condition had been identified were not more prone to increases in intraocular pressure than were those in which no such state had been registered. During the 8-month follow-up period, 1.5 % of the eyes required cataract surgery. No correlation has been observed between the number of dexamethasone injections, neither the time interval to re-injection, nor rises in intraocular pressure. Hence, it is unclear why the increases in intraocular pressure were less pronounced in the aforementioned German study [33] than in the North American Shasta trial [29, 30]. So, the findings of the latter study are more akin to those that have been reported for similarly afflicted eyes in Switzerland.

Two case studies in which anti-VEGF- and dexamethasone-based therapies were combined have been reported [34, 35]. In one of these — a retrospective interventional study — 33 RVO-afflicted eyes were intravitreally injected either with ranibizumab and then with dexamethasone, or with dexamethasone alone. The visual gains were more marked and were achieved more rapidly in the former than in the latter group [34]. In another prospective study, 34 RVO-afflicted eyes were intravitreally injected either with a combination of bevacizumab and dexamethasone, or with dexamethasone alone. The combined therapeutic regime proved to be more efficacious than the mono-therapeutic one with increase in visual acuity and reduction of central retinal thickness. The visual gains that were achieved persisted for longer periods of time [35]. This confirms the results of the Shasta trial in which combination therapy showed an extended duration of effect under Ozurdex if combined with anti-VEGF drugs. The risk of an increased IOP was higher in the combination therapy group, which may at least partially be explained by a selection bias [30].

Though the possibility of tachyphylaxis after repeated injections of intraocular corticosteroids has, meanwhile, been discussed for a decade [24, 36, 37], there is still no evidence for its existence after repeated use of intravitreal corticosteroids [26, 38, 39]. A rebound phenomenon, in contrast to tachyphylaxis, representing a more pronounced macular edema after loss of therapeutic effect has been reported [32, 40] and seems to be similar to that after repeated anti-VEGF injections in RVO [41, 42].

The efficacy of dexamethasone implants in cases with refractory macular edema under anti-VEGF therapy for retinal vascular disorders has been demonstrated [43]. Nevertheless, eyes pre-treated with anti-VEGF drugs, i.e. bevacizumab, seem to respond anatomically equally well to an additional therapy with triamcinolone or dexamethasone, although this does not obviously go along with an additional gain in visual function compared to the one in treatment-naïve eyes. The former, however, is prone to a more pronounced effect on the IOP of affected eyes, whereas the latter showed a moderately longer duration of effect [44].

The dexamethasone implant may be as effective as ranibizumab in treatment-naïve eyes with vision loss due to RVO [45, 46], requiring less injections for the well-known price of a medically well-controlled rise in IOP of more than 5 mmHg and cataract progression in nearly half of the eyes. On the other hand, change of treatment is less likely required after intravitreal dexamethasone than after anti-VEGF therapy [30, 47–50]. The dexamethasone implant may be used in cases in which the role of anti-VEGF agents has not been equally established, namely in ischemic retinopathies: its positive — though due to the ischaemic state, functionally limited — effect over 12 months in ischaemic RVO has prospectively been demonstrated [51, 52]. In individuals younger than 50 years with 50 % experiencing a three-line improvement in visual acuity with a mean of 1.8 implantations over 12 months was achieved. This is worth mentioning because these patients comprise the working-age group suffering mostly from the economic burden of frequent controls and reinjections [53].

Finally, the risk of complications, namely of an increase of IOP, does peak at 1 to 2 months after first treatment. Thereafter, no rise over time or with the number of injections was reported, although patients with pre-existing ocular hypertension and glaucoma may be exposed to a higher risk. An initial control of IOP will be needed within 4 weeks after implantation. Phakic patients have to expect cataract progression with the need of cataract surgery within roughly 1 year [54, 55].

As a therapeutic weapon against ME of various aetiologies, dexamethasone is assuming an increasingly elevated rank in the clinical arsenal, owing to its potency, the dose-consistency, the prolonged duration of action, and, compared to other corticosteroids, its favourable safety profile. However, few available prospective head-to-head comparisons and combinations with other treatment modalities allow as of yet one to precisely define the role of dexamethasone in clinical practice [15] (Table 1).

**Table 1:** Published evidence of long-term treatment with Ozurdex® in retinal vein occlusion

Ref. no. and first author (year of publication)	*n* (eyes) O: Dex C: Control	*n* (patients)	Study design R, P, PR, NR	Duration of follow-up period (months)	Number of patients with ME due to: C, B	Duration of ME before therapy (months)	Pre-treatment (N, A, T, L, O)	
[17] Coscas (2014)	O: 128	128	R	≥6	C: 58 B: 70	<6 in 62 patients	A: 39 L: 9 A + L: 8 O: 10	
[18] Mayer (2013)	Group 1: O: 38 Group 2: 3x bevacizumab followed by O: 26	64	P / NR	12	Group 1: C: 22 / B: 16 Group 2: C: 14 / B: 12	max. 4	Group 1: N Group 2: A: 26	
[22] Haller (2011)	1256 O (0.35 mg): 412 O (0.7 mg): 421 Sham: 423	1256	PR	12	n.r	>1.2	N	
[24] Joshi (2013)	O: 51	49	R	≥12	C: 28 / B: 23	C: 5.3 B: 5.5	C: N B: L: 6	
[27] Bezatis (2013)	O: 102	102	R	6	C: 48 B: 54	C: 10.6 B: 6	n.r.	
[29] Capone (2014)	O: 289	289	R	≥6	B:157 C:132	All: 18.4 B: 20.1 C: 16.4	A: 205 L: 112 N: 39	
[31] Querques (2013)	O: 33	32	R	≥6	C: 26 / B: 7	All: 16.9 C: 17.1 B: 16	A: 18 A + L: 2	
[32] Querques (2014)	O: 19	19	P	6	C: 13 / B:6	2.3	N	
[33] Schmitz (2014)	O: 324	342	R	8	n.r.	n.r.	n.r.	
[35] Singer (2012)	O: 34	33	P	6	C: 12 / B: 22	n.r.	A: 34	
[44] Ozkok	O: 35 T: 39 O: 30 /	74	R	≥12	B: O: 18, T: 20 C: O: 17, T: 19	n.r.	A: 74	
[45] Gado (2014)	Bevacizumab 30	60	PR	6	C = 60	n.r.	N	
[46] Matonti (2013)	O: 220	220	R	6	C: 89 B: 131	7.1	N	
Ref. no. and first Author (year of publication)	*n* (eyes) O: Dex C: Control	*n*(patients)	BCVA baseline (logMAR)	BCVA 90 days (logMAR)	CRT (μm) baseline	CRT (μm) 90 days	IOP max (mmHg), *n* = eyes	*n* (no. of eyes re-treated); no. of inj.; re-treatment interval (months)
[17] Coscas (2014)	O: 128	128	All patients: 0.42 BRVO: 0.44 CRVO: 0.41	n.r.	BRVO: 507.5 ± 158 CRVO: 629.4 ± 191.2	BRVO: 292.9 CRVO: 274.3	IOP >25 mm Hg *n* = 9	*n* = 128; −; 5.9 following the 1st inj. 8.7 following the 2nd
[18] Mayer (2013)	Group 1: O: 38 Group 2: 3x Bevaci-zumab followed by O: 26	64	Group 1: CRVO: 1.55 BRVO: 1.5 Group 2: CRVO: 1.7 BRVO: 1.45	n.r.	Group 1: CRVO: 604.4 ± 230.5 BRVO: 500.5 ± 106 Group 2: CRVO: 601.1 ± 252.5 BRVO: 469.8 ± 151.7	n.r.	Group 1: CRVO: *n* = 9 BRVO: *n* = 6 Group 2: CRVO: *n* = 6 BRVO: *n* = 5	Group 1: CRVO: *n* = 22; 2.4; 3.8 BRVO: *n* = 16; 1.8; 3.5 Group 2: CRVO: *n* = 14; 2.4; 3.2 BRVO: *n* = 12; 2; 3.7
[22] Haller (2011)	1256 O (0.35 mg): 412 O(0.7 mg): 421 Sham group: 423	1256	n.r.	n.r.	n.r.	n.r.	O (0.7 mg): *n* = 53 after 1st DEX inj. *n* = 65 after 2nd DEX inj.	O (0.35 mg/0.7 mg): 329; 2; 6 O (0.7 mg/0.7 mg): 341; 2; 6
[24] Joshi (2013)	O: 51	49	BRVO: 0.8 CRVO: 1.0	n.r.	BRVO: 451 ± 104 CRVO: 480 ± 174	BRVO: 280 * CRVO: 320 *	>25 mm Hg, *n* = 14	*n* = 19; 2; BRVO: 4.3 CRVO: 4.5
[27] Bezatis (2013)	O: 102	102	BRVO: 0.6 CRVO: 0.7	BRVO: 0.4 CRVO: 0.6	BRVO: 559 ± 209 CRVO: 740 ± 351	BRVO: 369 ± 126 CRVO: 455 ± 251	No ocular hyper-tension	BRVO *n* = 22; −; 4.4 CRVO: *n* = 24; −; 4.4
[29] Capone (2014)	O: 289	289	All patients: 1.0 BRVO: 0.9 CRVO: 1.2	n.r.	All patients: 438 ± 182 BRVO: 413 ± 149 CRVO: 469 ± 201	All patients ≦250 in 65.3 % BRVO: ≦250 in 66.0 % CRVO: ≦250 in 64.4 %	IOP >25 mm *n* = 95 IOP >35 mmHg *n* = 27	*n* = 186; M 3.2; 5.6
[31] Querques (2013)	O: 33	32	0.65	n.r.	636 ± 217	n.r.	*n* = 12	1st inj.: −; 4.7; 2; − 2nd inj.: −; 5.1; 2; −
[32] Querques (2014)	O: 19	19	0.5	0.54	762 ± 259	518 ± 251	24.3 mmHg, *n* = 5 >21 mmHg	-; −; 4.3
[33] Schmitz (2014)	O: 324	342	n.r.	n.r.	n.r.	n.r.	*n* = 62 >35 mmHg *n* = 6	-; 1.2; −
[35] Singer (2012)	O: 34	33	n.r.	n.r.	513	269	IOP ≥ 23 mmHg	*n* = 28; −;4.2
[44] Ozkok	O: 35 T: 39	74	BRVO: O: 0.44 T: 0.40 CRVO: O: 0.75 T: 0.71	n.r.	BRVO: O: 467.8 ± 125 T: 500 ± 78 CRVO: O: 518 ± 160 T: 550 ± 92	n.r.	*n* = 6 >21 mmHg O: *n* = 11 T: *n* = 15	BRVO: O: *n* = 18; 1.4; 7 T: 20; 2.1;5 CRVO: O: 17;1.6; 6.3 T: 19; 2.6; 4.6
[45] Gado (2014)	O: 30 / Bevaci-zumab 30	60	O group: 0.6 Bevaci-zumab group: 0.6	n.r.	O group: 548.53 ± 68.67 Bevacizu mab group: 544.13 ± 48.68	O group: 280 Bevaciz umab group: 283	O group: IOP > 21 mmHg *n* = 6	O group: *n* = 30; 2; 4
[46] Matonti (2013)	O: 220	220	All patients: 1.2 CRVO: 1.3 BRVO: 1.1	0.9	All patients: 641.86 ± 221.6 CRVO: 686 ± 247.1 BRVO: 612.4 ± 198.8	All patients: 362.5 ± 184.8	n.r.	*n* = 108; 2; 5.3

## Conclusion

To achieve satisfactory visual and anatomic outcomes after the intravitreal administration of dexamethasone implants, the re-injection interval needs to be considerably less than 6 months in many instances. To date, there are no indications that the long-term use of dexamethasone poses a safety hazard. Further evidence appertaining thereto has been recently furnished by a phase III trial (MEAD) in which diabetic ME was treated with intravitreal injections of dexamethasone implants. The findings revealed the lack of correlation between dexamethasone treatments per se and intermittent increases in intraocular pressure, or between the number of dexamethasone injections and rises in intraocular pressure per se [56]. In existing head-to-head trials in which the effects of anti-VEGF agents and dexamethasone have been directly compared, the interval of 6 months was, according to more recent experience, too protracted for the sustenance of the therapeutic effect, which can be maintained for approximately 4 months.

Published reports in which re-injections have been made after shorter intervals on an “as needed” basis are now available [26, 32]. Compared to anti-VEGF therapies, the burden of re-treatment is reduced. And in cases of chronic ME that are refractive to repeated anti-VEGF therapy, the response to dexamethasone is good. The future will reveal whether an early combined treatment strategy involving an anti-VEGF agent and dexamethasone elicits superior results to those that can be achieved using either of these drugs alone in terms of a long-term compensation of the underlying impaired vascular situation [34, 35].
